# Supplementary material for: Flexible ultrasound-induced retinal stimulating piezo-arrays for biomimetic visual prostheses
Source: Nat Commun. 2022 Jul 4;13:3853. doi: 10.1038/s41467-022-31599-4 (PMC9253314; doi:10.1038/s41467-022-31599-4)
Supplement: Supplementary file 1 — Supplementary Information [file 41467_2022_31599_MOESM1_ESM.pdf]

Supplementary Information for

Flexible Ultrasound-Induced Retinal Stimulating Piezo-arrays for Biomimetic Visual Prostheses

Laiming Jiang[1,2,3†*], Gengxi Lu[1,2†], Yushun Zeng[1,2†], Yizhe Sun[2], Haochen Kang[2], James Burford[1], Chen Gong[1,2], Mark S. Humayun[1,2,4], Yong Chen[3*], Qifa Zhou[1,2*]

[1]Roski Eye Institute, Department of Ophthalmology, Keck School of Medicine, University of Southern California, Los Angeles, California 90033, USA

[2]Department of Biomedical Engineering, Viterbi School of Engineering, University of Southern California, Los Angeles, California 90089, USA

[3]Epstein Department of Industrial and Systems Engineering, Viterbi School of Engineering, University of Southern California, Los Angeles, California 90089, USA

[4]Allen and Charlotte Ginsburg Institute for Biomedical Therapeutics, University of Southern California, Los Angeles, California 90089, USA

[†]Laiming Jiang, Gengxi Lu, and Yushun Zeng contributed equally to this work.

[*]Corresponding authors. Email: laiming_jiang@foxmail.com (L.J.); yongchen@usc.edu (Y.C.); qifazhou@usc.edu (Q.Z.)

This PDF file includes:

Supplementary Notes 1-6

Supplementary Tables 1-4

Supplementary Figs. 1-23

References

Supplementary Notes

Supplementary Notes 1: Fundamentals of electrical retinal stimulation.

Electrical stimulation of the nervous system has been studied for a long time. Electrode-based extracellular stimulation generally operates by injecting current into the tissue of interest by placing a single electrode or a group of electrodes nearby. For example, in electronic retina implants[1], the electrode array is placed close to the retina to form an electrochemical interface with saline. The current injected by the stimulation electrode passes through the retinal tissue to reach the return electrode. The electric current delivered to the extracellular area causes the charge on the cell membrane of the retinal neurons to redistribute. The action potential is initiated when the membrane depolarization exceeds the threshold.

The effects of stimulus pulse proximity between the electrode and neuron can be theoretically predicted using models of electrical stimulation. In a Rattay model[2], the extracellular electrode serves as an ideal point source, with a fixed distance from a uniform, infinitely long axon, which is useful to form a basic understanding of neural activation via extracellular electrodes. The polarity and extent of membrane polarization will change in response to the axonal stimulation with a monopolar electrode. In cathodic stimulation, negative charges accumulate on the outside of the membrane under the electrode, driving the intracellular movement of the positive charges from the adjacent compartments to this area, yielding strong membrane depolarization near the electrode and weak hyperpolarization away from the electrode. However, in anodic stimulation, strong hyperpolarization occurs in the membrane segment close to the electrode, and weak depolarization occurs at the distal end due to the reverse electric field.

For the electrical stimulation of the retina, a complex neural network, charge redistribution on the membrane of soma, axons, and dendrites will all contribute to the depolarization of the retinal neurons. The initial segment of the axon of retinal ganglion cells is located at the proximal end to the soma and contains high-density sodium channels. Extracellular stimulation of the retinal ganglion cells experimentally demonstrates that the axon initial segment possesses the smallest activation threshold, followed by other axonal sections and the soma, with the dendrites showing the maximum threshold[3]. At the subcellular level, an action potential initiated on one neuronal component may propagate to another neuronal component, thereby significantly affecting the time and space response dynamics of the cell[1].

Supplementary Notes 2: Acoustic impedance matching in ultrasonic transmission.

Acoustic interface that refers to the boundary between two materials with different acoustic impedances directly affects the transmission of ultrasound[4]. A certain amount of ultrasound energy is transmitted across the interface, and a certain amount of energy is reflected when acoustic waves enter an acoustic interface with normal incidence. For an ultrasound receiver, the reflection of ultrasound off the surface is inevitable because of the acoustic impedance mismatch between the ultrasound media (e.g., ultrasound gel, tissue) and the device. The power reflected can be written as[5]

$$\frac{P_{\mathrm{reflected}}}{P_{\mathrm{total}}}=\left(\frac{Z_{\mathrm{d}}-Z_{\mathrm{m}}}{Z_{\mathrm{d}}+Z_{\mathrm{m}}}\right)^{2} \tag{S1}$$

wherein Z_{d} and Z_{m} are the acoustic impedance of the device and media, respectively. For example, the acoustic impedances for piezoelectric ceramics and crystals are generally 20-30 MRayls, for 1-3 composite are generally 10-15 Mrayls, but the acoustic impedance of the ultrasound gel or tissue is relatively low (~ 1.5 MRayls). A useful method to reduce the reflection is to add an acoustic matching layer between the piezoelectric layer and ultrasound media. Ideal acoustic impedances of a matching layer (Z_{ml}) can be evaluated as[6]

$$Z_{\mathrm{ml}}=(Z_{\mathrm{d}}Z_{\mathrm{m}})^{1/2} \tag{S2}$$

Building a 1-3 piezoelectric composite composed of piezoelectric ceramics/crystals and a polymer is also an effective approach to lower the acoustic impedance, thereby diminishing the power loss caused by the reflection. In addition, the acoustic impedance and electromechanical coupling performance of a composite could also be tailored via varying the volume fraction and aspect ratio of piezoelectric pillars.

Supplementary Notes 3: The frequency selection.

First, ultrasound technology has been applied for over 20 years and has an excellent safety record. The usual range of medical ultrasound for procedural guidance is 1-18 MHz[7]. Therefore, for bio-implantable applications, the selection of ultrasonic frequency is preferred to be in this range, similar to other medical ultrasound devices.

Second, the wavelength of ultrasound directly affects its resolution (resolution $\propto \lambda$) because the focusing is wavelength-dependent (please refer to **Supplementary Notes 5**). High-frequency

ultrasound has shorter wavelengths for higher resolution. Our receiving piezoelectric array is with 32 elements and each element size is $1 \times 1\ mm^2$. The gap between the two elements is 0.5 mm. To enhance the resolution of the acoustic beam without affecting the operation of the adjacent piezoelectric element, the wavelength of ultrasound should be less than 0.5 mm. For example, the wavelength 3.3-MHz ultrasound is ~ 0.47 mm, is possible to meet the resolution requirements.

Third, the attenuation of ultrasound energy is linearly dependent on the ultrasound frequency. For example, the ultrasound has a ~ 0.5-1 dB cm^{-1} MHz^{-1} acoustic attenuation coefficient in tissue[8]. High frequency will cause high attenuation, thereby reducing transmission efficiency.

Therefore, combining the above aspects (the usual range of medical ultrasound, wavelength, and attenuation), 3.3-3.5 MHz ultrasound that combines high resolution with low attenuation but in the usual medical range was selected in this work.

Supplementary Notes 4: Acoustic field simulation in Field II.

The acoustic field of the ultrasound transducer was simulated by Field II, which is a program based on the calculation of spatial impulse responses as suggested by Tupholme[9] and Stepanishen[10]. The concept is similar to the impulse response in any linear system and assumes a homogeneous and bounded medium where the pressure is small enough to ensure linear wave propagation. Therefore, single-element and multi-element arrays can be processed because the response is simply a superposition of the responses from different elements that are correctly phase aligned. This approach can be illustrated using Huygens' principle, where the impulse response is computed from the sum of all the spherical surface waves from the aperture region S as[11]

$$h(\vec{r}1,t)=\int \frac{\delta(t-\frac{|\vec{r}1-\vec{r}2|}{c})}{2\pi|\vec{r}1-\vec{r}2|}dS \tag{S3}$$

wherein $|\vec{r}1-\vec{r}2|$ is the distance from the transducer at position $\vec{r}2$ to the field point at $\vec{r}1$, $\delta(\mathrm{t})$ is the Dirac delta function, and c is the acoustic velocity.

In addition, it is notable that the impulse responses are dependent on the relative position between the transmitter and the receiver, hence the term spatial impulse response. It can be used to calculate any type of linear ultrasound field. The emitted acoustic pressure field $p(\vec{r}1,t)$ is written as[11]

$$p(\vec{r}1,t)=\rho_0\frac{\partial_{\mathrm{v}}(t)}{\partial \mathrm{t}}\times h(\vec{r}1,t) \tag{S4}$$

where ρ_0 is the density of the medium and $\partial v(t)/\partial t$ is the acceleration of the front face of the transducer.

Supplementary Notes 5: Resolution calculation of the focused single transducer.

The spherical focusing confirmation of the transducer can not only enhance the magnitude of the acoustic excitation but also effectively improve the lateral resolution of the ultrasound beam. The lateral resolution ($R_{lateral}$) is mainly dependent on device geometry, which can be theoretically estimated by[12]

$$R_{lateral}=\lambda \times f\# \qquad \text{(S5)}$$

The f-number is used to describe the ratio between the focal length and the aperture size. In our work, the 3.3-MHz focused single transducer has an aperture size of a diameter of 30 mm and a focal length of 25 mm. The lateral resolution ($R_{lateral}$) is calculated to be ~ 390 μm.

Supplementary Notes 6: Calcium imaging of retina tissue

Calcium ions generate a variety of intracellular signals that determine a large variety of functions in virtually every cell type in biological organisms[13,14]. In the nervous system, calcium ions preserve and, perhaps, even extend their high degree of versatility because of the complex morphology of neurons. Calcium imaging techniques are generally employed to obtain the activities of neuronal populations. They have been an increasingly popular experimental method that enables the monitoring of targeted neural populations in alive and behaving animals. This microscopy method offers a signal for the actual neural population activity.

In this work, calcium imaging was used to study the response of retinal neuron activity to the electrical stimulation of the F-URSP. The retina was dissected from the Ai95(RCL-GCaMP6f)-D transgenic mice, which possess genetically encoded calcium indicators that facilitate imaging activity of genetically defined neuronal populations and are useful for long-term, high-sensitivity imaging[15].

Supplementary Tables

Supplementary Table 1. Comparison of ultrasound waves and electromagnetic (EM) waves.

Specification	Ultrasound waves	EM waves	References
Wavelength	150-1500 μm (@ 1-10 MHz)	4.55-45 mm (@ 1-10 GHz)	-
FDA regulatory limit	720 mW cm^{-2}	10 mW cm^{-2}	Sonmezoglu et al[16]; Lin et al[17]
Attenuation in tissue	~ 0.5-1 dB cm^{-1} MHz^{-1}	~ 10-12 dB cm^{-1} (@ 2 GHz)	Thimot et al[18]
Security in cybercommunications	High	Low (potential cyber-vulnerabilities)	-
Focusing*	Small, diameter ~ 0.15-1.5 mm (@ 1-10 MHz)	Large, diameter ~ 4-40 mm (@ 1-10 GHz)	-
Wave velocity	~ 330 m s^{-1} (@ Air)<br>~ 1540 m s^{-1} (@ Tissue)<br>~ 1500 m s^{-1} (@ Water)	~ 3×10^8 m s^{-1}	-
Speed of scan (frame-rate/PRF)**	0-5 kHz	120 Hz (Argus® II System (Second Sight, Sylmar, CA)	Piech et al[19]; Zhou et al[20]
Pattern	Beam Pattern	Encoding	-
Stimulation threshold	~ 0.8 V	~ 0.8 V	Zhou et al[20]
Secondary effects	Cavitation, and thermal and mechanical effects	Sleep disturbances, headache, fatigue, and depression	Shankar et al[21]
Waveform type	Longitudinal and Transverse waves	Transverse wave	-
Medium	Yes	No	-
Carcinogenicity	No	Yes	-

* The focusing is wavelength dependent. For example, the diameter of -6 dB focusing region for ultrasound is $D = \lambda \times f\#$, where λ is wavelength and f-number is used to describe the ratio between the focal length and the aperture size of transducer[22]. If the $f\#$ value is 1, the focusing region is theoretically close to the wavelength. ****** The systems could produce a broad set of clinically relevant stimulation currents, pulse widths and pulse repetition frequencies (PRFs); these were sufficient to elicit highly repeatable potentials in the nerve. For example, David K. Piech et al. reported an ultrasound-based implantable neural stimulator with used PRF up to 5 kHz in burst[19]. In our work, the PRF can be up to 1kHz in burst.

Supplementary Table 2. Parameters for the bulk PZT ceramic and manufactured PZT/epoxy 1-3 composite.

Parameters	PMN-PT crystal	PMN-PT/epoxy 1-3 **composite** (this work)
Piezoelectric constant d_{33}	1140 pC N^{-1}	1050 pC N^{-1}
Electromechanical coupling coefficient k_t	0.6	0.84
Relative free permittivity $\varepsilon_{33}^{T}/\varepsilon_0$ (1kHZ)	5500	2940
Relative clamped permittivity $\varepsilon_{33}^{S}/\varepsilon_0$	700	420
Dielectric loss tan δ	0.006	0.005
Piezoelectric voltage coefficient g_{33}	23.4 $\times 10^{-3}$ V m N^{-1}	40.3 $\times 10^{-3}$ V m N^{-1}
Thickness h	N/A	180 μm
Density ρ	8100 kg m^{-3}	5260 kg m^{-3}
Acoustic impedance Z_a	30.1 MRayls	10.6 MRayls

The piezoelectric constant d_{33} was characterized by a Belincourt-type d_{33} meter (YE2730A, APC Products, Inc). The capacitance and dielectric loss were measured by an inductance-capacitance-resistance (LCR) digital bridge machine (QuadTech). Impedance spectrums were characterized by an impedance analyzer (Agilent, 4294A). Detailed parameters were calculated by the following equations[23]

$$\varepsilon^{\mathrm{T}}/\varepsilon_0 = \frac{C^{\mathrm{T}}h}{\varepsilon_0 A}, \tag{S6}$$

$$g_{33} = \frac{d_{33}}{\varepsilon_{33}^{\mathrm{T}}}, \tag{S7}$$

$$k_{\mathrm{t}} = \sqrt{\frac{\pi}{2}\frac{f_{\mathrm{r}}}{f_{\mathrm{a}}}\tan\left(\frac{\pi}{2}\frac{f_{\mathrm{a}}-f_{\mathrm{r}}}{f_{\mathrm{a}}}\right)}, \tag{S8}$$

$$c = f \cdot \lambda = 2f_{\mathrm{r}} \cdot h, \tag{S9}$$

$$Z_{\mathrm{a}} = \rho \cdot c. \tag{S10}$$

Supplementary Table 3. Some studies of electrical stimulation for the retina, peripheral nerves, sciatic nerves, etc.

Energy Delivery Method	Stimulating Electrode Position	Electrical parameters	References
Electromagnetic (EM) waves	Epiretinal, human in vivo	39 μA	Humayun et al[24]
Wired electrodes	Epiretinal, isolated chicken retina	0.6V, 1.2 V, 1.8 V	Stett et al[25]
Wired electrodes	Subretinal, cat in vivo	50 μA	Sachs et al[26]
Wired electrodes	Epiretinal stim, cat in vivo	14 nC	Hesse et al[27]
Wired electrodes	Transchoroidal, normal and retinal degenerate rat	7.2 nC (normal retina)<br>12.9 nC (degenerate retina)	Humayun et al[28]
Coil inductive	Rat sciatic nerve	2 V	Lee et al[29]
Radio Frequency (RF) link	Ex vivo rat sciatic nerve	1.2 V	Khalifa et al[30]
Ultrasound link	Rat sciatic nerve	3 V	Piech et al[19]
Ultrasound link	Rat peripheral nerves	10-20 nC	Chen et al[31]
Ultrasound link	Rat abdomen	3.3 V	Luo et al[32]
Ultrasound link	Isolated rat retina	1-2 V	This work

Supplementary Table 4. Acoustic and thermal specifications of human eye tissues

Eye tissues	Density (kg m^{-3})	Sound speed (m s^{-1})	Heat capacity at constant pressure (J kg^{-1} K^{-1})	Thermal conductivity (W m^{-1} K^{-1})	Attenuation (dB cm^{-1} MHz^{-1})
Water	1000	1500	4178	0.62	0
Cornea	1062	1586	4178	0.58	0.78
Vitreous	1005	1532	3999	0.6	0.01
Lens	1076	1647	3000	0.40	1.19
Retina	1034	1538	3680	0.57	1.15

Acoustic and thermal specifications of human eye tissues were obtained from the published literature[33].

Supplementary Figures

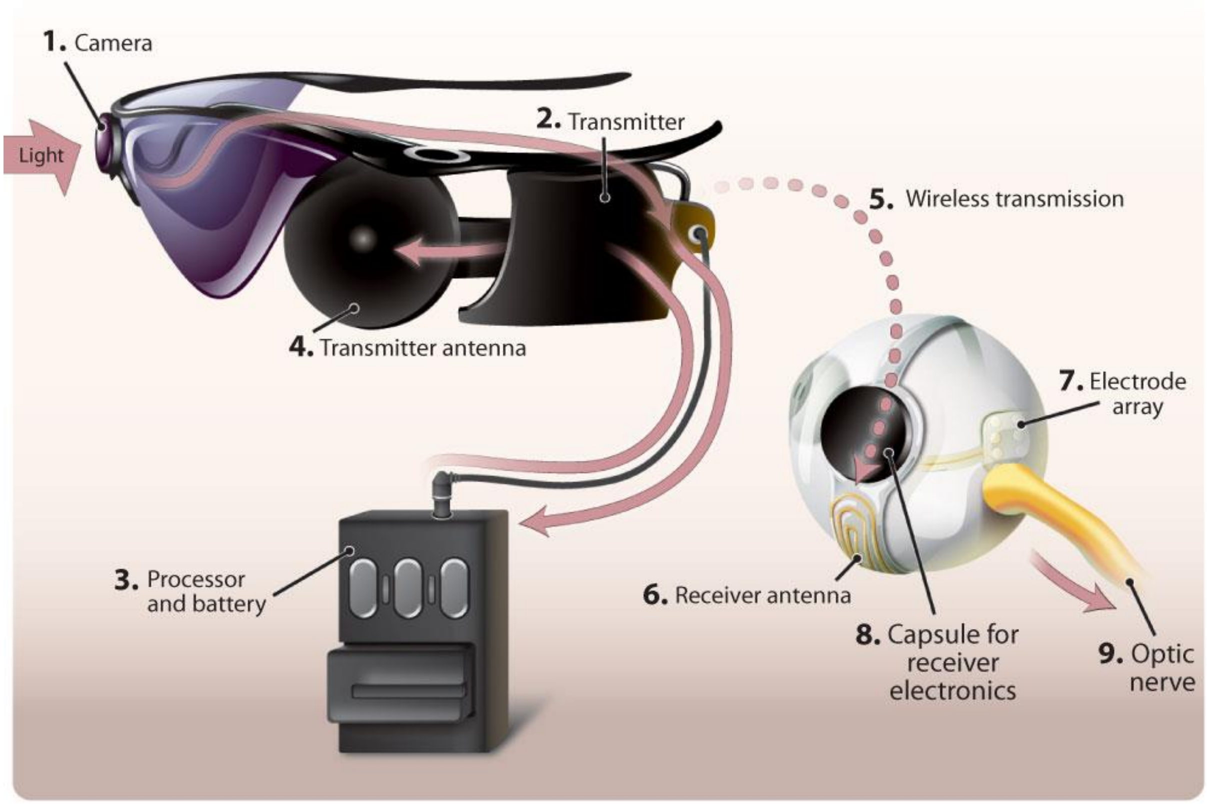

Supplementary Figure 1 | Argus® II System (Second Sight, Sylmar, CA). The implant consists of a receiving coil for receiving information and power from the external components of the Argus® II System, an electronics package that is secured to the outside of the eyeball using a standard scleral band and that drives stimulation of the electrodes and an electrode array. A minimally invasive surgery is required to implant the array on the retina. From [Zrenner, E. Fighting blindness with microelectronics. *Sci. Transl. Med*. 5, 210-216 (2013)]. Reprinted with permission from AAAS[34].

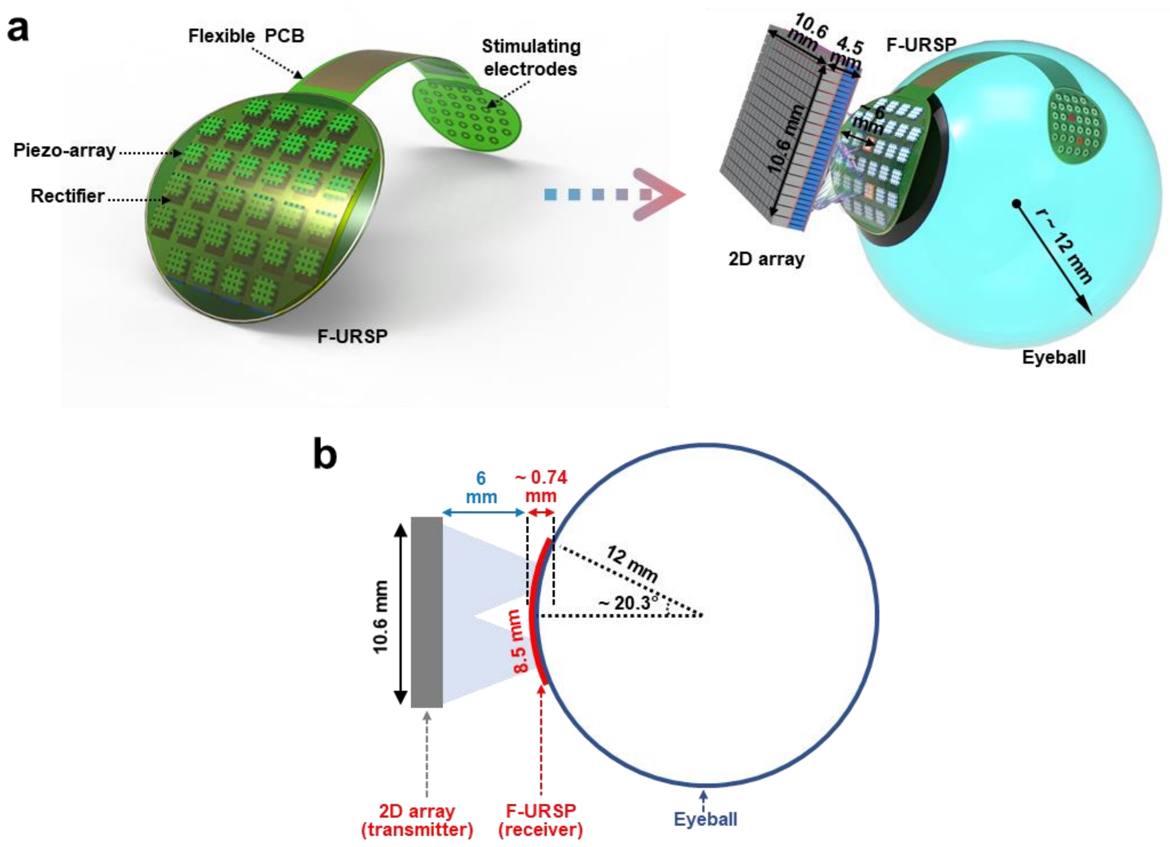

Supplementary Figure 2 | Schematic diagram showing the design structure of the F-URSP for biomimetic visual prostheses. a, Schematic diagram showing the structure of the device. The design refers to the size of the human eyeball (radius ~ 12 mm) for future implant applications. **b,** Schematic diagram showing the size of the device on the eyeball. The difference of the distance between the outermost element and the center element can be compensated by adjusting the direction and focus depth of the acoustic beams by using a 2D array transmitter (please refer to **Supplementary Fig. 17**).

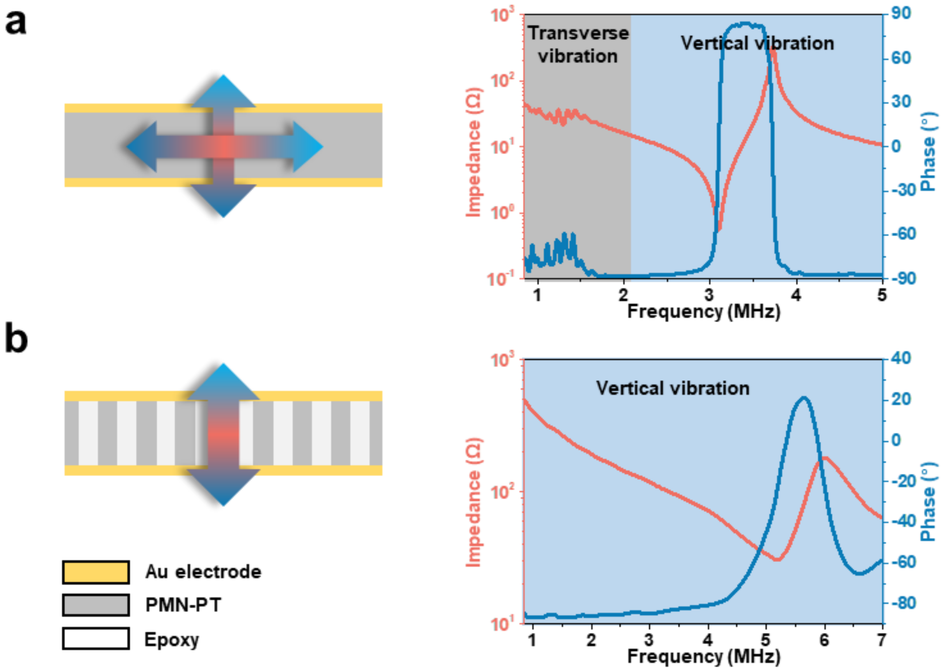

Supplementary Figure 3 | The vibration mode comparison between bulk PMN-PT and PMN-PT/Epoxy 1-3 composite. a,**b**, Vibration schematics (left) and electrical impedance and phase angle (right) of (**a**) bulk PMN-PT and (**b**) fabricated PMN-PT/Epoxy 1-3 composite. Compared to bulk PMN-PT, the epoxy in the 1-3 composite confines the transverse vibration of the PMN-PT pillars and concentrates the energy on the longitudinal vibration. That is, the PMN-PT pillars in the 1-3 composite vibrate in the fundamental length longitudinal 33-mode with higher efficiency determined by the piezoelectric coupling factor k_{33}. Source data are provided as a Source Data file.

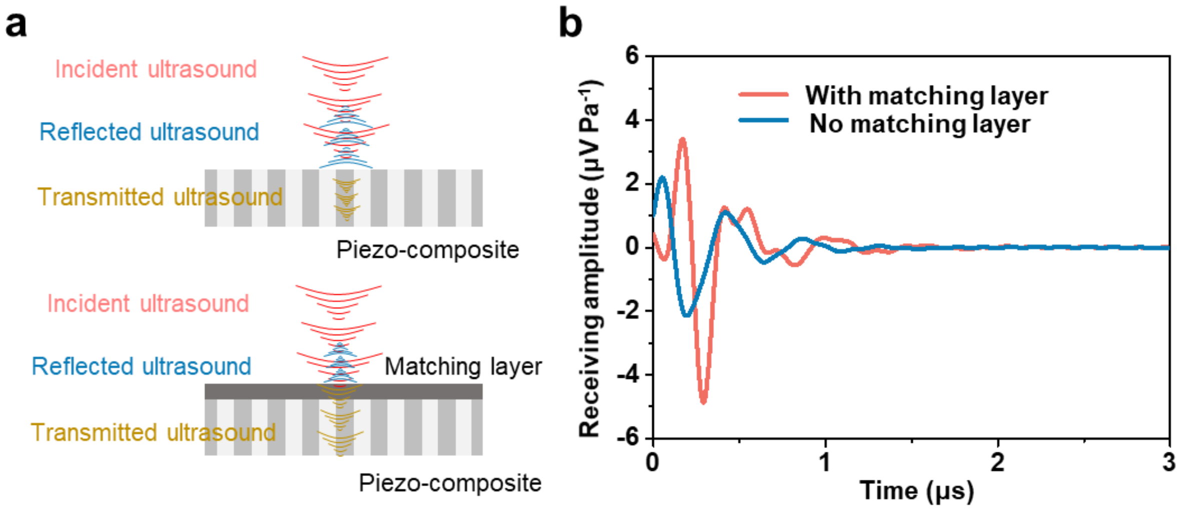

Supplementary Figure 4 | Comparing the receiving sensitivity of the piezo-element with and without matching layer. a, Schematic diagram showing the ultrasound at the interface of the piezoelectric component with and without a matching layer. **b**, Simulated receiving sensitivity of the piezo-element with and without a matching layer by using PiezoCAD. A matching layer can compensate for the acoustic impedance mismatch between the composite and ultrasound gel and thus improve ultrasound energy transmission. Source data are provided as a Source Data file.

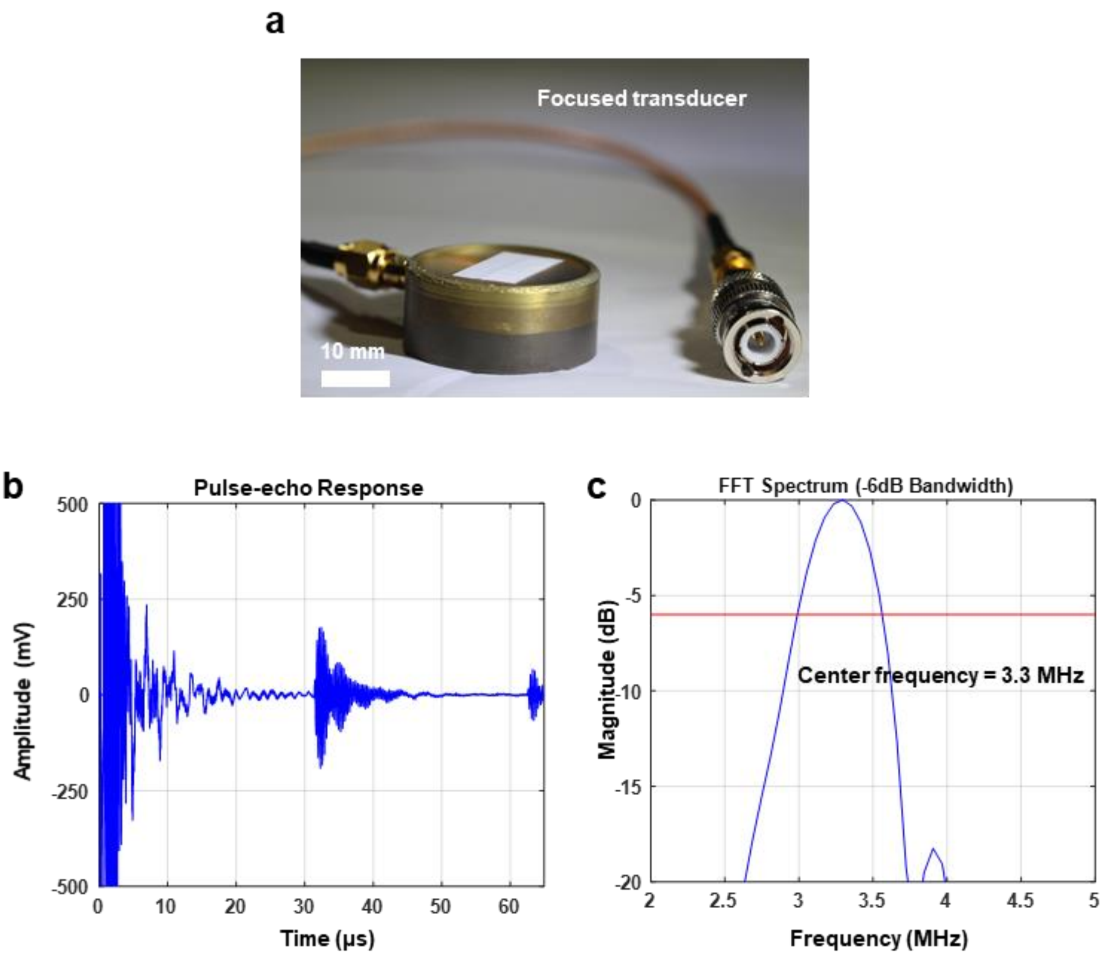

Supplementary Figure 5 | Pulse-echo response of the 3.3-MHz focused probe. **a**, Photograph showing the focused transducer. **b**, Pulse-echo measurement of the focused transducer. **c**, FFT spectrum of the pulse-echo response. The focused transducer used for ultrasound delivery is designed and fabricated in our laboratory by using a focused PZT ceramic disc with a diameter of 30 mm and a focus length of 25 mm. It shows a center frequency of 3.3 MHz, a bandwidth of 17%. Source data are provided as a Source Data file.

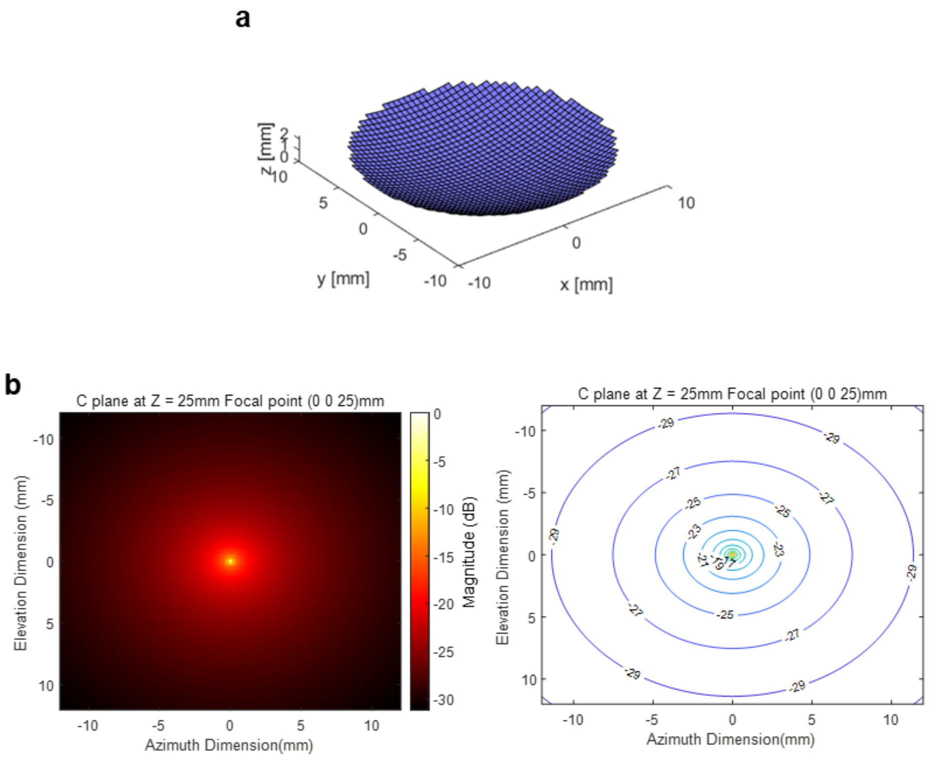

Supplementary Figure 6 | Ultrasound field simulation of the 3.3-MHz focused probe. **a**, Schematic diagram showing the focused geometric architecture of the probe. **b**, A simulated ultrasound field at c plane emitted by the focused probe using Field II software package, showing a focused ultrasound area. The results should be noted that the -6 dB (energy attenuated by half) lateral resolution of the emitted 3.3-MHz ultrasound beam is ~ 390 μm near the focus point, which is below one element size (1 mm × 1 mm) of the receiving piezo-array, thus ensuring the less impact on adjacent elements when an element is excited. Receiving maps depending on one single element are shown in **Fig. 4d**.

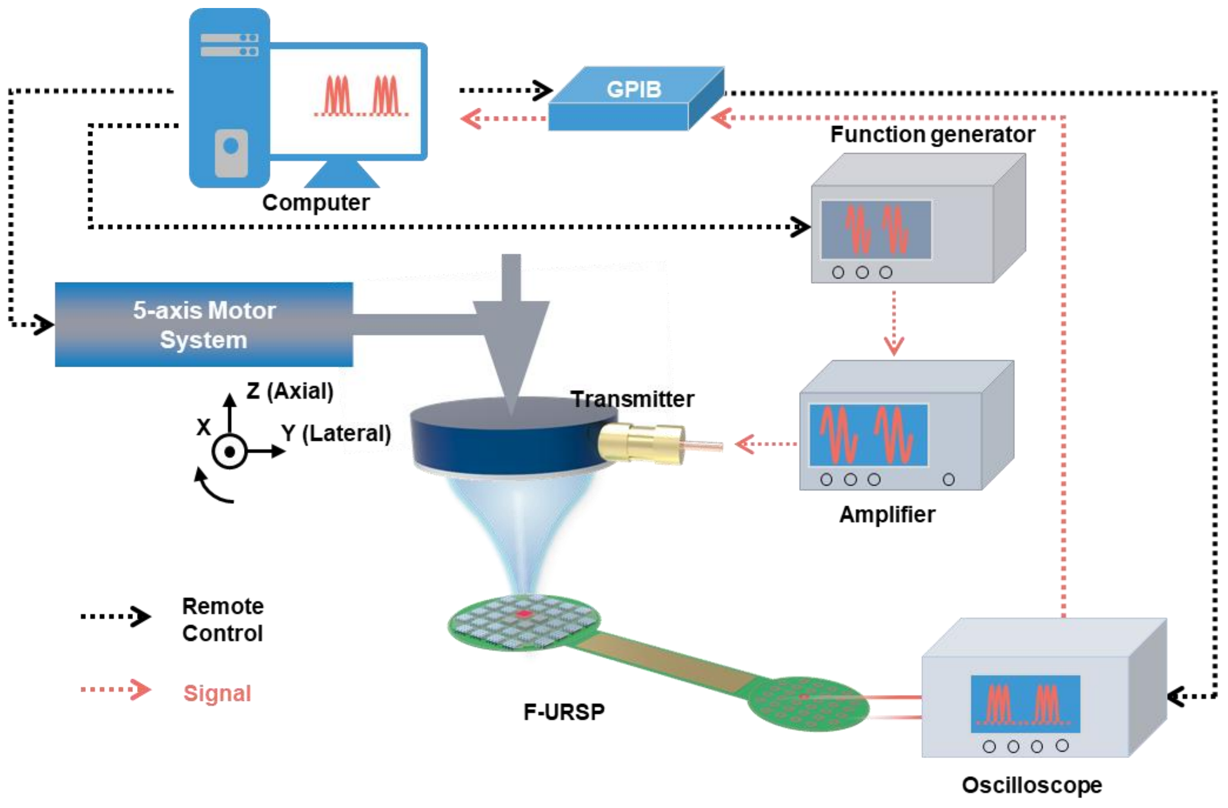

Supplementary Figure 7 | Output test platform. The US-induced electrical outputs of the F-URSP were measured using a multifunctional US testing platform. US transducers as the external acoustic sources were mounted on a 5-axis motorized stage (Opto-Sigma). F-URSP as an US receiver was placed in front of the transducer. Degassed US gel (EcoVue US Gel, HR Pharmaceuticals, Inc.) was applied as the coupling medium between transducer and biomimetic visual prostheses for transmitting US waves. The US transducer was driven via a tone sine-wave burst, which was generated by a function generator (AFG3252C, Tektronix) and then amplified by 40 dB with an amplifier (75A250A, AR RF/Microwave Instrumentation). An oscilloscope (TDS 5052, Tektronix) with an internal resistance of 1 MΩ was used to measure the output voltages generated by the device. The acoustic pressure of the ultrasonic transmitter was measured in the water tank by a hydrophone probe (HGL-1000, ONDA, Inc., Sunnyvale, CA, USA).

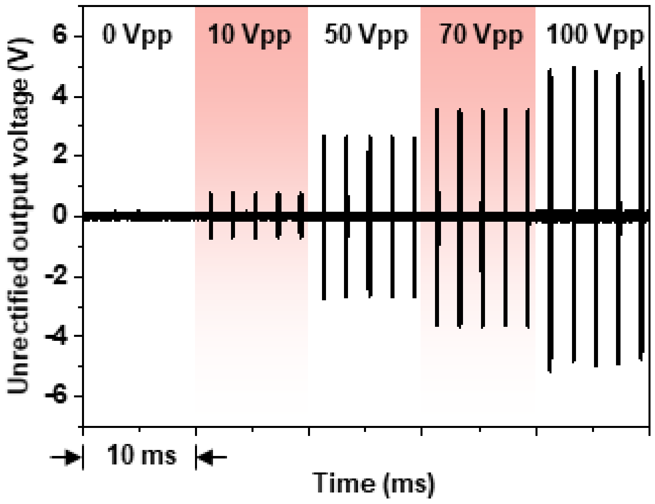

Supplementary Figure 8 | Unrectified voltages of the device under the different trigger voltages. The output voltage can be flexibly adjusted by the trigger voltage. Source data are provided as a Source Data file.

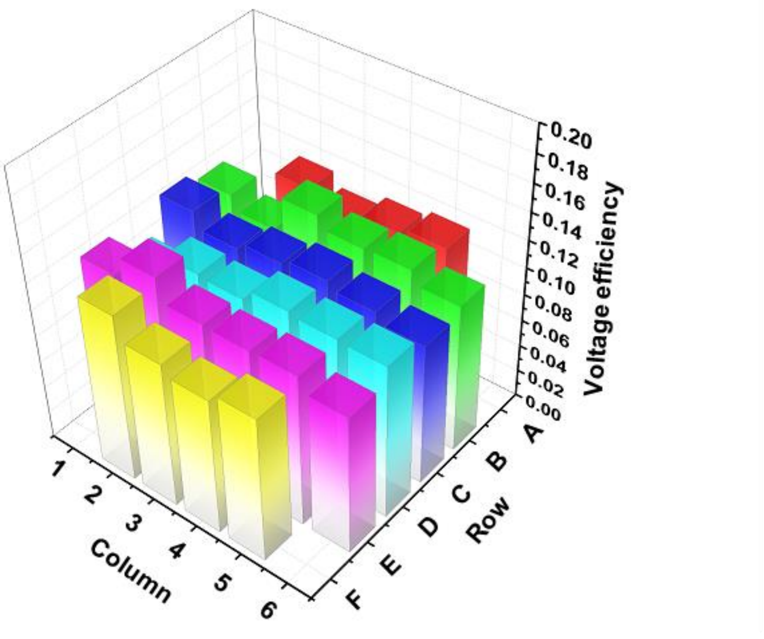

Supplementary Figure 9 | The voltage efficiency (output voltage/input voltage, Vpp/Vpp) variation of the 32 piezoelectric elements. The voltage efficiency has a mean value of 11.3% with the standard deviation of 0.75%, demonstrating good uniformity. Source data are provided as a Source Data file.

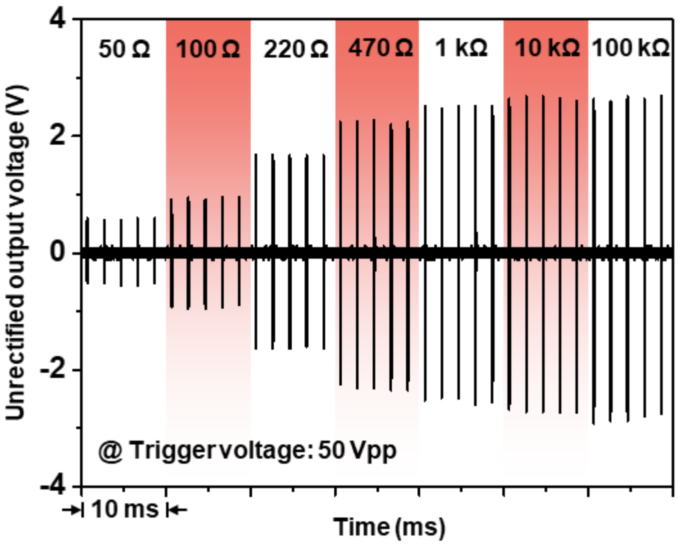

Supplementary Figure 10 | Unrectified output voltage magnitudes of the device under various load resistors. As the resistance increases, the output voltage increases progressively and then saturates at higher external loads. Source data are provided as a Source Data file.

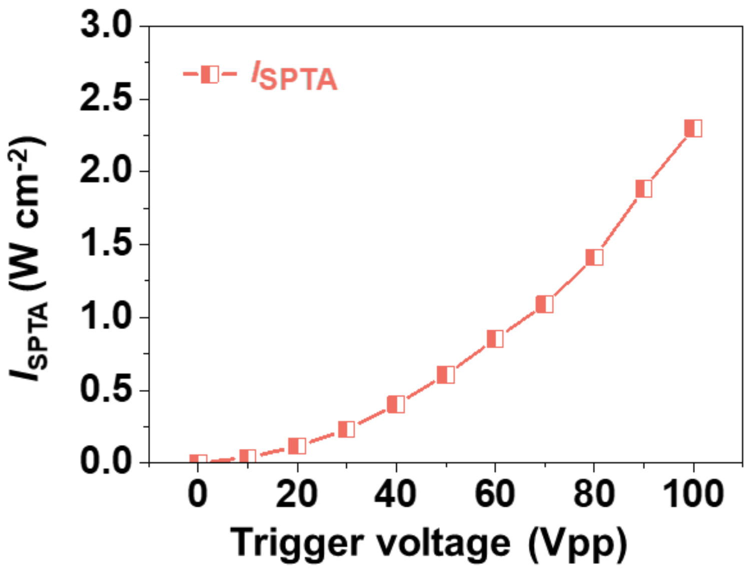

Supplementary Figure 11 │ Spatial peak temporal average intensity (I_{SPTA}) at the face of receiving array alongside the trigger voltage. The I_{SPTA} represents the focus area intensity at the face of receiving array and is calcultaed by I_{SPTA} = NPP^2/2ρc × Duty cycle, where NPP is the negative peak pressure, ρ is the density (~ 1000 kg m^{-3}), and c is sound speed (~ 1540 m s^{-1}) in the medium. The duty cycle used is 10%. Since the acoustic beam is focused, the intensity at the face of receiving array (focus area) is, on average, 5.3 times the intensity at the face of transducer. Source data are provided as a Source Data file.

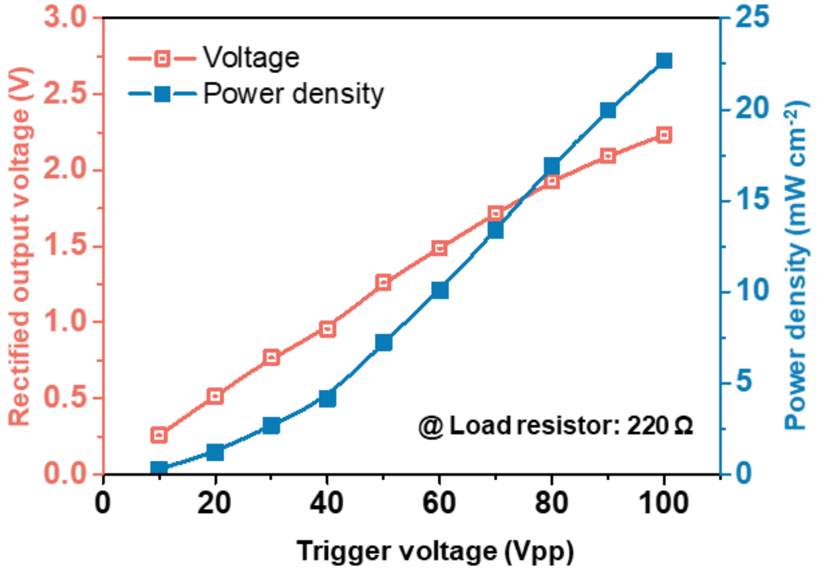

Supplementary Figure 12 | Output voltage and corresponding power density of one piezo-element under load resistor of 220 Ω and various trigger voltages. The results show that the outputs can be flexibly adjusted to reach a power density up to 22.6 mW cm^{-2}. Source data are provided as a Source Data file.

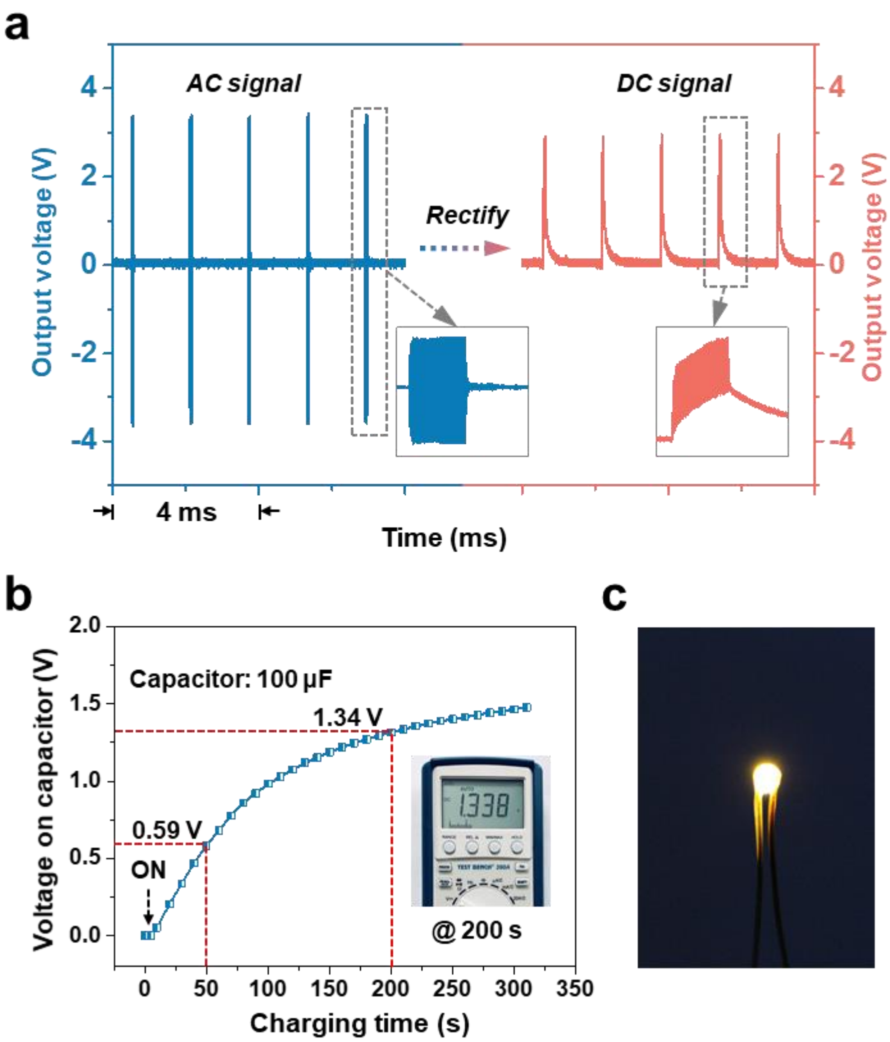

Supplementary Figure 13 | Charging rate characterization of the F-URSP. a, Output voltage magnitudes of the device before (left) and after (right) rectification. **b**, The charging time dependence of voltage on a 100 μF capacitor; the inset shows the measured voltage (≈ 1.34 V) charged for 200 s. **c**, Photographs showing a commercial LED lit up by the electricity stored in the capacitor. Source data are provided as a Source Data file.

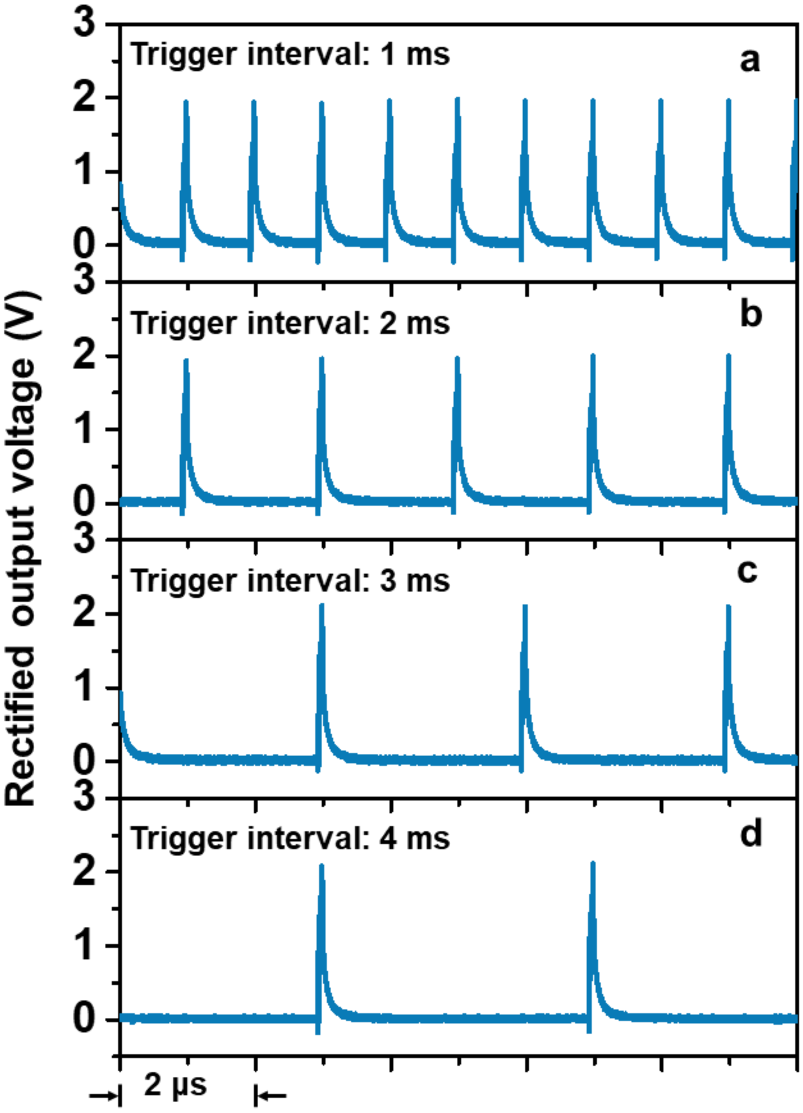

Supplementary Figure 14 | Output voltage magnitudes of the device under the excitation of trigger signals with different trigger intervals. **a**, 1ms . **b**, 2 ms. **c**, 3 ms. **d**, 4 ms. Source data are provided as a Source Data file.

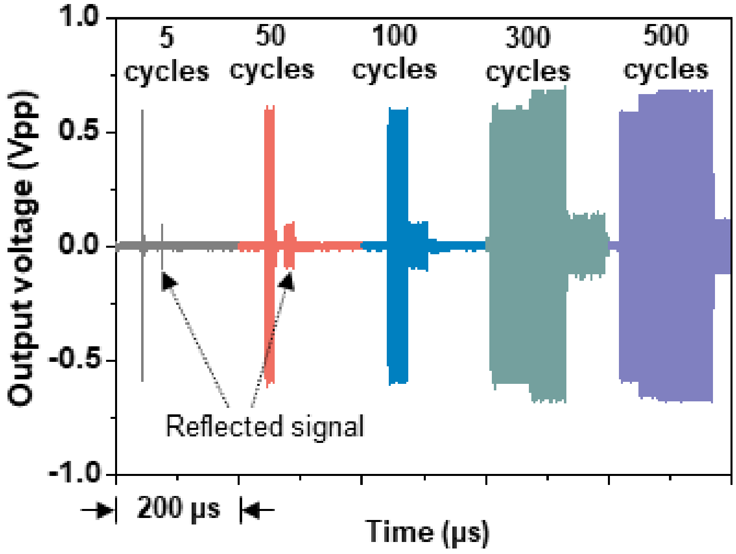

Supplementary Figure 15 | Unrectified output voltage waveforms of the device under a burst mode with different trigger repetition cycles. The results demonstrate that ultrasound-induced energy can be flexibly tunable by changing the duty cycle. Source data are provided as a Source Data file.

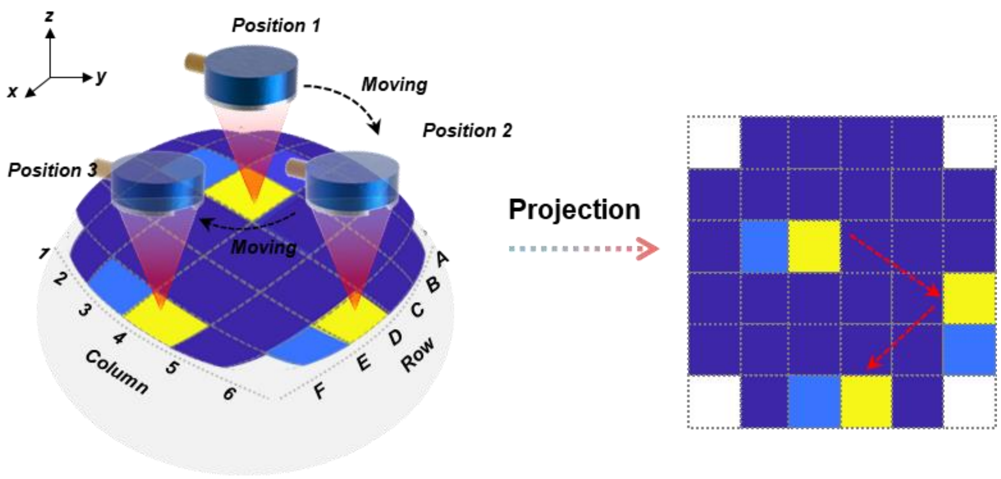

Supplementary Figure 16 | Pattern reconstruction schematic and acoustic field simulation of a single focused transducer. The schematic diagram shows the reconstruction of the pattern by the moving single focus transducer.

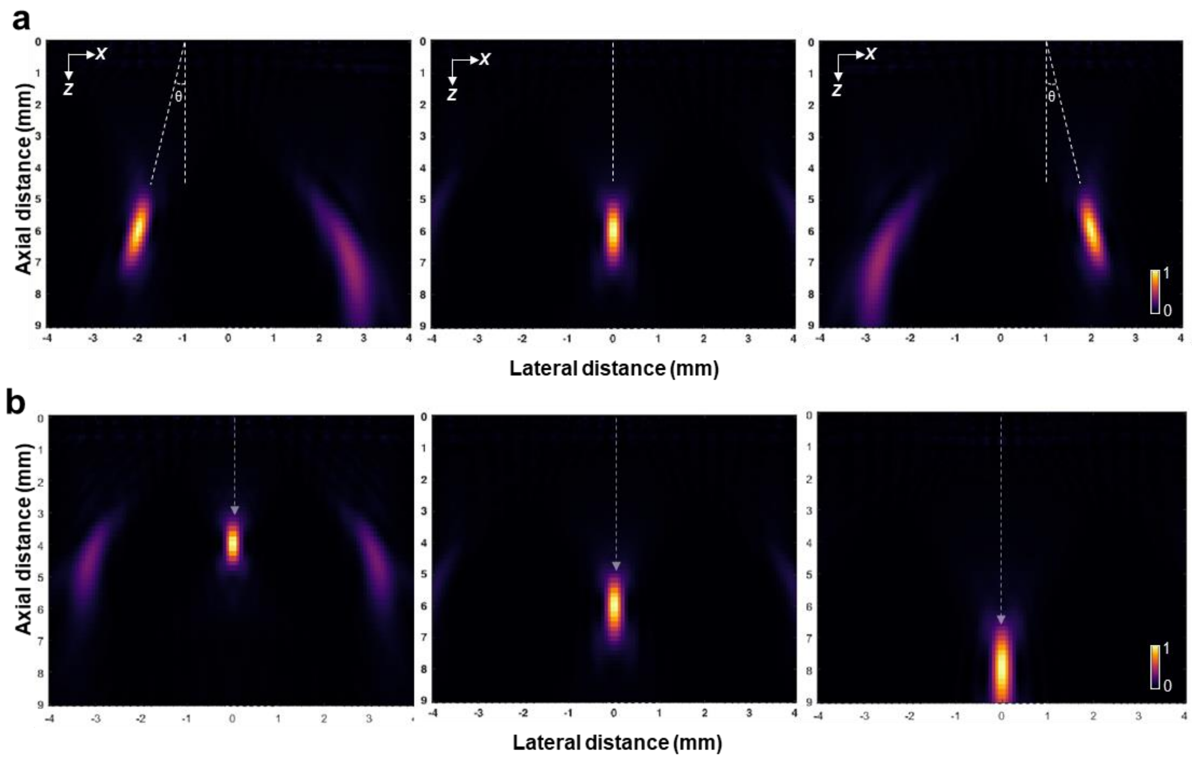

Supplementary Figure 17 | Simulated ultrasonic fields demonstrating ultrasound beam steering and focusing of a 2D array transducer. a, Simulated steering of ultrasonic beamlines. **b,** Simulated focusing at different depths of ultrasonic beamlines. The multi-element 2D array transducer exhibits the scalability and programmability to allow steering and focusing at different depths of ultrasonic beamlines in the viewing direction by modifying the transmitting amplitude and phase of each element. The color bar (a,b) indicates the normalized acoustic pressure. The maximum value is 0.5 MPa.

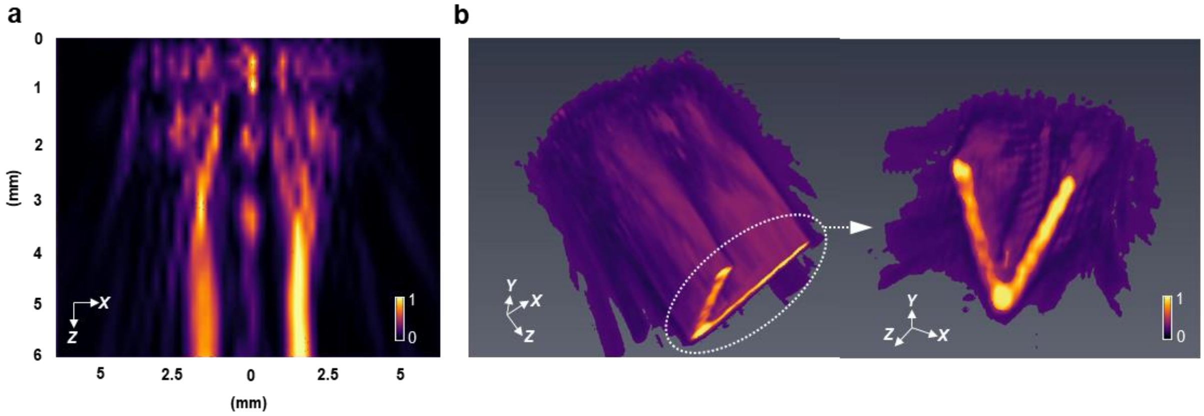

Supplementary Figure 18 | Simulated ultrasonic field. **a**, Simulated acoustic intensity distribution at the *X*-*Z* plane of a "V" pattern. **b**, Simulated 3D acoustic intensity distribution of a "V" pattern emitted by a 2D array transducer. The color bar (a,b) indicates the normalized acoustic pressure. The maximum value is 0.5 MPa.

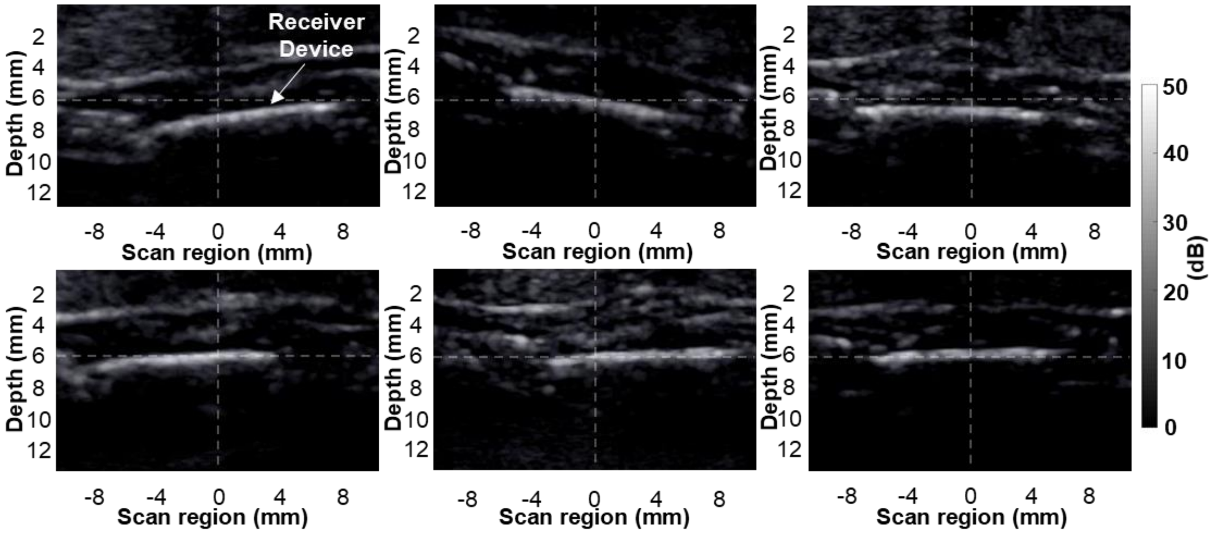

Supplementary Figure 19 | The alignment of the "transducer-receiver" calibrated by ultrasound imaging. Additionally, the transducer is not only used for the emission of programmable acoustic fields, but also for ultrasound imaging to evaluate the alignment of the transmitter and receiver. Depending on the results of ultrasound imaging, the relative position of the transmitter that is fixed on the 3D adjustable mount can be flexibly adjusted to align it with the receiver prior to the emission of acoustic fields.

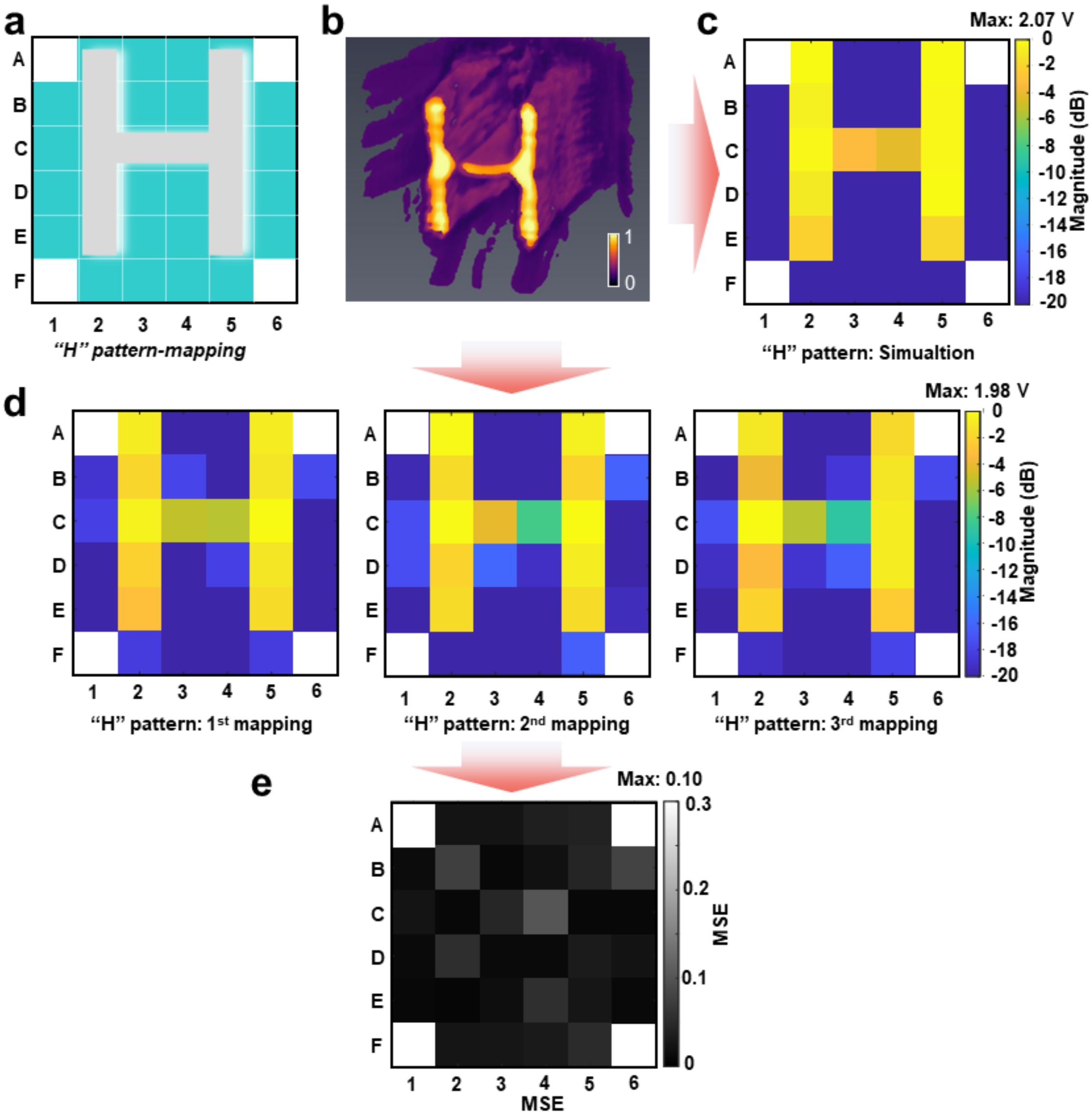

Supplementary Figure 20 | Mean Squared Error (MSE) per pixel between a simulated image reconstruction and the measured results. (a) Schematic diagram showing the reconstruction of a "H" pattern. (b) Simulated acoustic field and image outputs. The color bar indicates the normalized acoustic pressure. The maximum value is 0.5 MPa. (c) Simulated image outputs. The color bar indicates the magnitude distribution of ultrasound-induced piezoelectric potentials. The maximum value is 2.07 V. (d) Measured image outputs of multiple tests. The color bar indicates the magnitude distribution. The maximum value is 1.98 V. (e) MSE per pixel between simulated outputs and the measured results. The MSE values below 0.1. Source data are provided as a Source Data file.

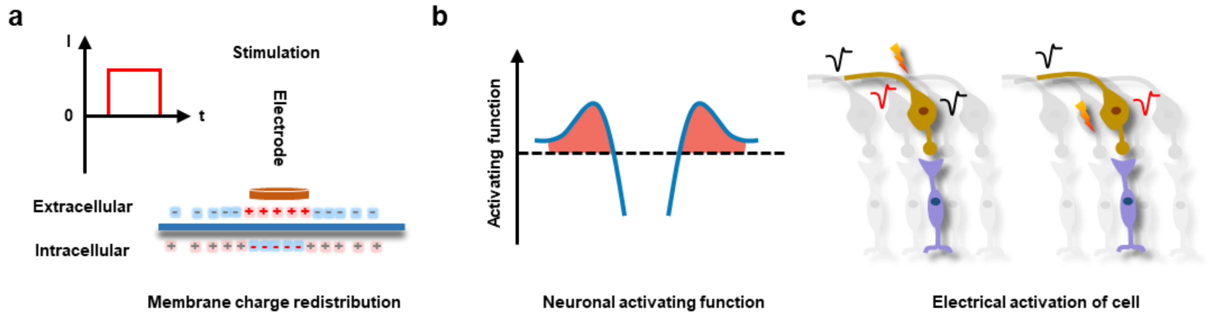

Supplementary Figure 21 | Schematically illustrates the fundamentals of electrical stimulation to the retina. a, Redistribution of membrane charge induced by an electrical pulse. **b,** Neuronal activating function. Shaded areas represent the activated membrane segments. **c,** Schematic diagram of the direct electrical activation of a retinal cell (left). Membrane depolarization initiated at the soma of the retinal ganglion cell (yellow) propagates to the axon (right).

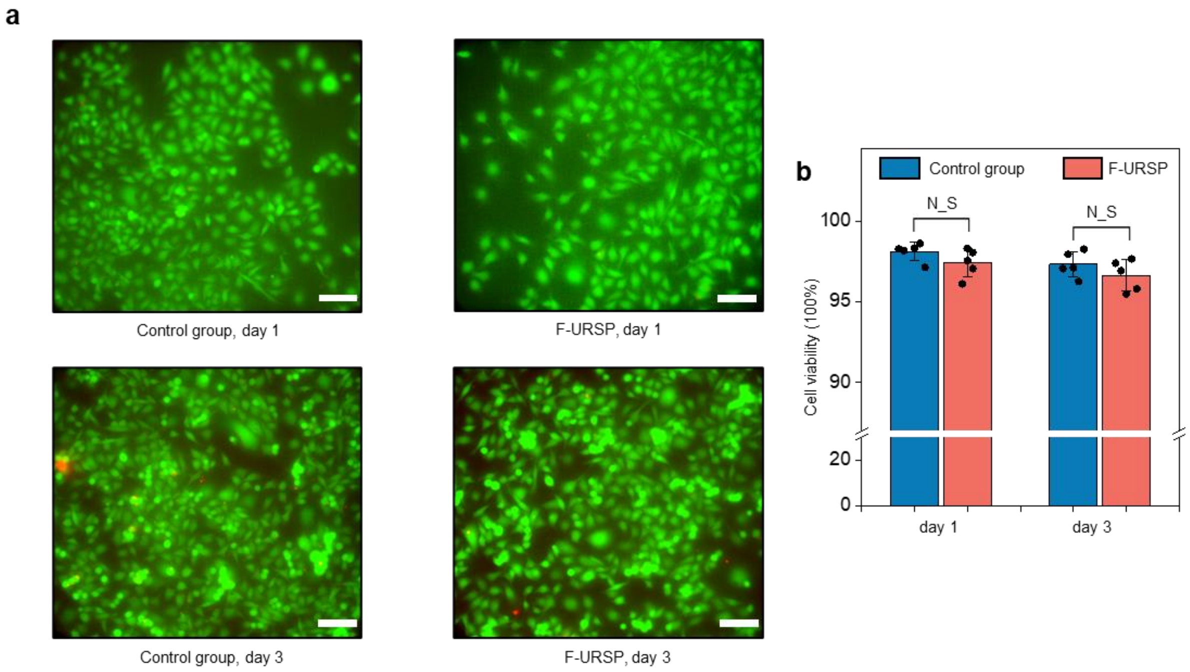

Supplementary Figure 22 | Cytocompatibility of F-URSP. a, Cytocompatibility test: live and dead cell staining of the PC-3 seeded on the petri-dish substrate and the F-URSP on day 1 and day 3, respectively. The viable cells were stained in green, and the dead cells were stained in red. Scale bar: 100 μm. **b,** Cell viability of PC-3 cell lines seeded on the petri dish substrate and F-URSP after 1 (p = 0.978 > 0.01) and 3 (p = 0.977 > 0.01) days of culture. N = 5 independent cell line samples. Data were presented as mean ± SD. The error bars represent the standard deviations (SD). N_S: None Significant Difference. Source data are provided as a Source Data file.

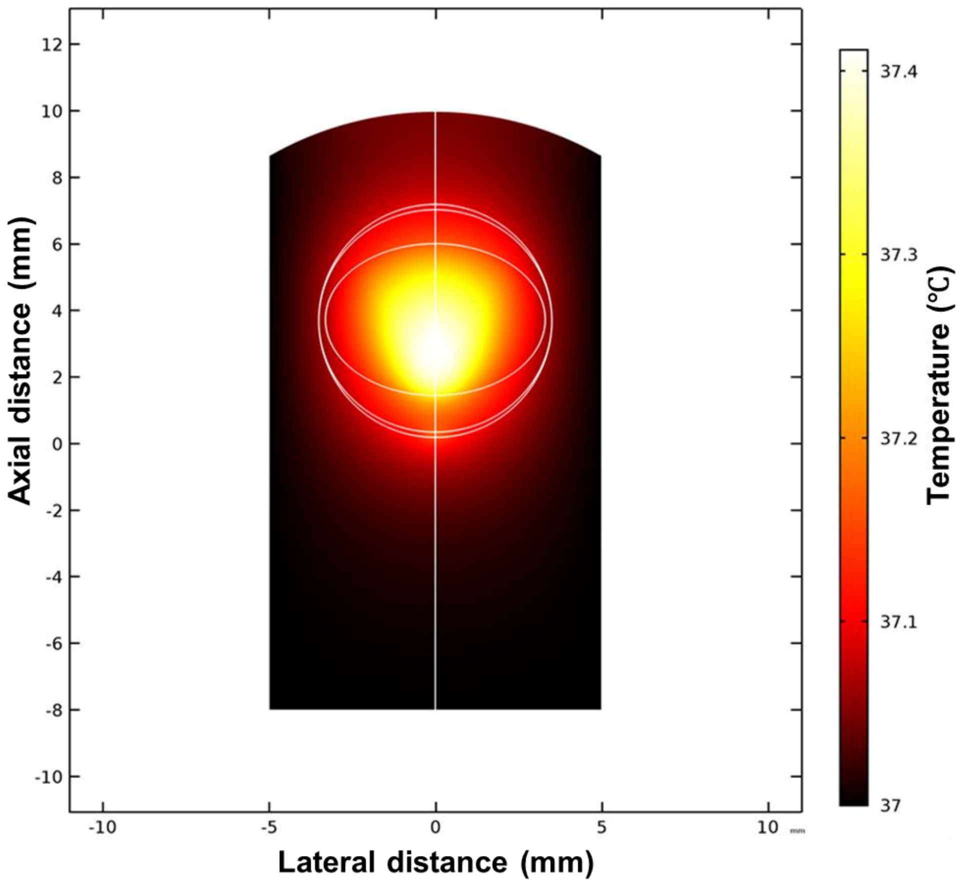

Supplementary Figure 23 | FEA simulation of the temperature increase of human eye tissue exposed to ultrasound. Simulation (parameters: 10% duty cycle, 1000 cycles per pulse, 50 Vpp) indicates that the ultrasound-induced temperature increase is less than 0.5 °C. Most of the increased heat occurs within the lens because of the high acoustic attenuation of the lens (Detailed parameters are listed in the following **Supplementary Table 4**). The temperature increase in eye tissue was experimentally measured (~ 0.4 °C) by inserting the implant into an excised porcine eyeball (Sierra Medical Science, Inc., Whittier, CA, USA) exposed to ultrasound at the same time-averaged acoustic intensity. Consequently, the piezo-array is ultrasonically safe to operate with average ultrasound intensity stimulation.

References

1 Yue, L., Weiland, J. D., Roska, B. & Humayun, M. S. Retinal stimulation strategies to restore vision: Fundamentals and systems. *Prog. Retin. Eye Res.* **53**, 21-47 (2016).

2 Rattay, F. Analysis of models for external stimulation of axons. *IEEE Trans. Biomed. Eng.* **33**, 974-977 (1986).

3 Tsai, D. *et al.* Responses of retinal ganglion cells to extracellular electrical stimulation, from single cell to population: model-based analysis. *PloS One* **7**, e53357 (2012).

4 Jiang, L., Yang, Y., Chen, Y. & Zhou, Q. Ultrasound-induced wireless energy harvesting: From Materials Strategies to functional applications. *Nano Energy*, 105131 (2020).

5 Christensen, D. *The wave equation and its solutions*. (John Wiley, New York, 1988).

6 Zhou, Q., Lam, K. H., Zheng, H., Qiu, W. & Shung, K. K. Piezoelectric single crystal ultrasonic transducers for biomedical applications. *Prog. Mater Sci.* **66**, 87-111 (2014).

7 Bhatia, A. & Peng, P. In *Essentials of Pain Medicine* 725-736 (Elsevier, 2018).

8 Oglat, A. A. *et al.* Chemical items used for preparing tissue-mimicking material of wall-less flow phantom for doppler ultrasound imaging. *J. Med. Ultrasound* **26**, 123 (2018).

9 Tupholme, G. E. Generation of acoustic pulses by baffled plane pistons. *Mathematika* **16**, 209-224 (1969).

10 Stepanishen, P. R. Transient radiation from pistons in an infinite planar baffle. *J. Acous. Soc. Am.* **49**, 1629-1638 (1971).

11 Jensen, J. A. Simulation of advanced ultrasound systems using Field II. In *Proc. 2004 2nd IEEE International Symposium on Biomedical Imaging: Nano to Macro* 636-639 (IEEE, 2004).

12 Jiang, L. *et al.* Fabrication of a (K,Na)NbO_3-based lead-free 1-3 piezocomposite for high-sensitivity ultrasonic transducers application. *J. Appl. Phys.* **125**, 214501 (2019).

13 Grienberger, C. & Konnerth, A. Imaging calcium in neurons. *Neuron* **73**, 862-885 (2012).

14 Berridge, M. J., Lipp, P. & Bootman, M. D. The versatility and universality of calcium signalling. *Nat. Rev. Mol. Cell Bio.* **1**, 11-21 (2000).

15 Dana, H. *et al.* Thy1-GCaMP6 transgenic mice for neuronal population imaging in vivo. *PloS One* **9**, e108697 (2014).

16 Sonmezoglu, S., Fineman, J. R., Maltepe, E. & Maharbiz, M. M. Monitoring deep-tissue oxygenation with a millimeter-scale ultrasonic implant. *Nat. Biotechnol.* **39**, 855-864 (2021).

17 Lin, J. C. A new IEEE standard for safety levels with respect to human exposure to radio-frequency radiation. *IEEE Antenn. Propag. Mag.* **48**, 157-159 (2006).

18 Thimot, J. & Shepard, K. L. Wirelessly powered implants. *Nat. Biomed. Eng.* **1** (2017).

19 Piech, D. K. *et al.* A wireless millimetre-scale implantable neural stimulator with ultrasonically powered bidirectional communication. *Nat. Biomed. Eng.* **4**, 207-222 (2020).

20 Zhou, D. D., Dorn, J. D. & Greenberg, R. J. The Argus® II retinal prosthesis system: An overview. In *Proc. 2013 IEEE International Conference on Multimedia and Expo Workshops (ICMEW)* (IEEE, 2013).

21 Shankar, H., Pagel, P. S. & Warner, D. S. Potential adverse ultrasound-related biological effects: a critical review. *J. Am. Soc. Anesth.* **115**, 1109-1124 (2011).

22 Zhou, Q. F., Lau, S. T., Wu, D. W. & Shung, K. Piezoelectric films for high frequency ultrasonic transducers in biomedical applications. *Prog. Mater Sci.* **56**, 139-174 (2011).

23 Meeker, T. ANSI/IEEE standard on piezoelectricity. *IEEE Trans. Ultrason. Ferroelectr. Freq. Control* **43**, 717-772 (1996).

24 Humayun, M. S. *et al.* Visual perception in a blind subject with a chronic microelectronic retinal prosthesis. *Vision Res.* **43**, 2573-2581 (2003).

25 Stett, A., Barth, W., Weiss, S., Haemmerle, H. & Zrenner, E. Electrical multisite stimulation of the isolated chicken retina. *Vision Res.* **40**, 1785-1795 (2000).

26 Sachs, H. *et al.* The Evaluation of Subretinal Stimulation by Film–Electrode Arrays Suitable for Chronic Human Experiments in Animal Models. *Invest. Ophth.Vis. Sci.* **45**, 4206-4206 (2004).

27 Hesse, L., Schanze, T., Wilms, M. & Eger, M. Implantation of retina stimulation electrodes and recording of electrical stimulation responses in the visual cortex of the cat. *Graef. Arch. Clin. Exp.* **238**, 840-845 (2000).

28 O'Hearn, T. *et al.* Electrical stimulation of normal and retinal degenerate (rd) isolated mouse retina. *Invest. Ophth.Vis. Sci.* **43**, 4467-4467 (2002).

29 Lee, B. *et al.* An Implantable Peripheral Nerve Recording and Stimulation System for Experiments on Freely Moving Animal Subjects. *Sci. Rep.* **8** (2018).

30 Khalifa, A. *et al.* The Microbead: A Highly Miniaturized Wirelessly Powered Implantable Neural Stimulating System. *IEEE T. Biomed. Circ. S.* **12**, 521-531 (2018).

31 Chen, P. *et al.* Ultrasound-driven electrical stimulation of peripheral nerves based on implantable piezoelectric thin film nanogenerators. *Nano Energy* **86** (2021).

32 Luo, Y. S. *et al.* Ultrasonic Power/Data Telemetry and Neural Stimulator With OOK-PM Signaling. *IEEE T. Circuits-II* **60**, 827-831 (2013).

33 Nabili, M., Geist, C. & Zderic, V. Thermal safety of ultrasound-enhanced ocular drug delivery: A modeling study. *Med. Phys.* **42**, 5604-5615 (2015).

34 Zrenner, E. Fighting blindness with microelectronics. *Sci. Transl. Med.* **5**, 210-216 (2013).
