# Supplementary material for: Flexible ultrasound-induced retinal stimulating piezo-arrays for biomimetic visual prostheses
Source: Nat Commun. 2022 Jul 4;13:3853. doi: 10.1038/s41467-022-31599-4 (PMC9253314; doi:10.1038/s41467-022-31599-4)
Supplement: Supplementary file 3 — Reporting Summary [file 41467_2022_31599_MOESM3_ESM.pdf]

nature portfolio

Corresponding author(s): Laiming Jiang, Yong Chen, Qifa Zhou

Last updated by author(s): May 27, 2022

Reporting Summary

Nature Portfolio wishes to improve the reproducibility of the work that we publish. This form provides structure for consistency and transparency in reporting. For further information on Nature Portfolio policies, see our Editorial Policies and the Editorial Policy Checklist.

Statistics

For all statistical analyses, confirm that the following items are present in the figure legend, table legend, main text, or Methods section.

n/a	Confirmed	
☐	☒	The exact sample size (*n*) for each experimental group/condition, given as a discrete number and unit of measurement
☐	☒	A statement on whether measurements were taken from distinct samples or whether the same sample was measured repeatedly
☒	☐	The statistical test(s) used AND whether they are one- or two-sided<br>*Only common tests should be described solely by name; describe more complex techniques in the Methods section.*
☒	☐	A description of all covariates tested
☒	☐	A description of any assumptions or corrections, such as tests of normality and adjustment for multiple comparisons
☐	☒	A full description of the statistical parameters including central tendency (e.g. means) or other basic estimates (e.g. regression coefficient) AND variation (e.g. standard deviation) or associated estimates of uncertainty (e.g. confidence intervals)
☒	☐	For null hypothesis testing, the test statistic (e.g. *F*, *t*, *r*) with confidence intervals, effect sizes, degrees of freedom and *P* value noted<br>*Give P values as exact values whenever suitable.*
☒	☐	For Bayesian analysis, information on the choice of priors and Markov chain Monte Carlo settings
☒	☐	For hierarchical and complex designs, identification of the appropriate level for tests and full reporting of outcomes
☒	☐	Estimates of effect sizes (e.g. Cohen's *d*, Pearson's *r*), indicating how they were calculated

Our web collection on statistics for biologists contains articles on many of the points above.

Software and code

Policy information about availability of computer code

Data collection	An oscilloscope (TDS 5052, Tektronix) with an internal resistance of 1 MΩ was used to measure the output voltages generated by the device. Custom Matlab (Matlab R2019b, Mathworks, Inc.) code was used to program the external 2D transducer via Verasonics release 4.2 system (Verasonics, Inc.). Receiving signals and ultrasonic imaging were recorded in the same Verasonics system.
Data analysis	Data was analysed by using Matlab R2019b, Mathworks, Inc. Origin 9.0 was used to plot the data.

For manuscripts utilizing custom algorithms or software that are central to the research but not yet described in published literature, software must be made available to editors and reviewers. We strongly encourage code deposition in a community repository (e.g. GitHub). See the Nature Portfolio guidelines for submitting code & software for further information.

Data

Policy information about availability of data

All manuscripts must include a data availability statement. This statement should provide the following information, where applicable:

- Accession codes, unique identifiers, or web links for publicly available datasets
- A description of any restrictions on data availability
- For clinical datasets or third party data, please ensure that the statement adheres to our policy

The dataset that supports the plots within this paper and other findings of this study are available from the corresponding author upon reasonable request.

Field-specific reporting

Please select the one below that is the best fit for your research. If you are not sure, read the appropriate sections before making your selection.

☒ Life sciences ☐ Behavioural & social sciences ☐ Ecological, evolutionary & environmental sciences

For a reference copy of the document with all sections, see nature.com/documents/nr-reporting-summary-flat.pdf

Life sciences study design

All studies must disclose on these points even when the disclosure is negative.

Sample size	The study was carried out with three isolated retinas dissected from three Ai95(RCL-GCaMP6f)-D transgenic male mice (at 12-14 weeks of age) (The Jackson Laboratory). Wireless stimulation was confirmed in these three isolated retinas via calcium imaging. See Methods, 'Study of Living Retina Stimulation' for details.
Data exclusions	No data were excluded from the analyses.
Replication	Experimental findings were reproducible in the same subject. Wireless stimulation with an device was replicated across three isolated retina.
Randomization	Tests were selected randomly. The key effect shown was controllable stimulation, evidenced by calcium imaging replicated across three isolated retina.
Blinding	Blinding was not relevant to our study, as there were no subjective elements to the assessment of the system capabilities.

Reporting for specific materials, systems and methods

We require information from authors about some types of materials, experimental systems and methods used in many studies. Here, indicate whether each material, system or method listed is relevant to your study. If you are not sure if a list item applies to your research, read the appropriate section before selecting a response.

Materials & experimental systems

n/a	Involved in the study	
☒	☐	Antibodies
☐	☒	Eukaryotic cell lines
☒	☐	Palaeontology and archaeology
☐	☒	Animals and other organisms
☒	☐	Human research participants
☒	☐	Clinical data
☒	☐	Dual use research of concern

Methods

n/a	Involved in the study	
☒	☐	ChIP-seq
☒	☐	Flow cytometry
☒	☐	MRI-based neuroimaging

Eukaryotic cell lines

Policy information about cell lines

Cell line source(s)	Prostate cancer cell (PC-3) lines were obtained from American Type Culture Collection (ATCC) by Dr. Nan Sook Lee.
Authentication	Cell line was authenticated using short tandem repeat (STR) analysis by the University of Arizona Genetics Core.
Mycoplasma contamination	The cell line was tested negative for mycoplasma.
Commonly misidentified lines (See ICLAC register)	No commonly misidentified cell line was used.

Animals and other organisms

Policy information about studies involving animals; ARRIVE guidelines recommended for reporting animal research

Laboratory animals	Ai95(RCL-GCaMP6f)-D transgenic male mice (at 12-14 weeks of age).
Wild animals	This study did not involve wild animals.
Field-collected samples	This study did not involve field-collected samples.

Ethics oversight	The Institutional Animal Care and Use Committee of the University of Southern California approved all protocols (protocol number 20978) and procedures. All animal research conforms to the National Institutes of Health (NIH) GUIDELINES as outlined in the Guide for the Care and Use of Laboratory Animals.

Note that full information on the approval of the study protocol must also be provided in the manuscript.
